# A Novel Piezoelectric-Assisted Non-Surgical Periodontal Treatment: A Prospective Case Series

**DOI:** 10.3390/dj11070178

**Published:** 2023-07-20

**Authors:** Roberto Rotundo, Lorenzo Marini, Mauro Carere, Cinzia Trezza, Giovanni Marras, Michele Nieri, Andrea Pilloni

**Affiliations:** 1 Periodontology Unit, Vita-Salute San Raffaele University, 20132 Milan, Italy; 2 Section of Periodontology, Department of Dental and Maxillofacial Sciences, Sapienza, University of Rome, 00161 Rome, Italy; lorenzo.marini@uniroma1.it (L.M.); dott.cinziatrezza@gmail.com (C.T.); andrea.pilloni@uniroma1.it (A.P.); 3 Private Practice, 00187 Rome, Italy; mauro.carere@gmail.com; 4 Private Practice, Castletown, Isle of Man IM9 1AB, UK; marrasgio@gmail.com; 5 Department of Surgery and Translational Medicine, University of Florence, 50121 Florence, Italy; michelenieri@gmail.com

**Keywords:** periodontal debridement, periodontal diseases, periodontitis, root planing, therapeutics

## Abstract

The purpose of this study was to evaluate the clinical efficacy of a non-surgical periodontal treatment using a piezoelectric power-driven device with a novel insert. Plaque index (PlI), bleeding on probing (BoP), probing depth (PD), recession depth (Rec) and clinical attachment level (CAL) were assessed at 6 weeks, 3 months and 6 months. Furthermore, tooth mobility and furcation involvement were recorded and chewing discomfort and dental hypersensitivity were evaluated. Eighteen stage I to IV periodontitis patients providing 437 teeth and 2622 sites in total were analyzed. At six weeks, CAL gain (0.4; *p* < 0.0001), PD reduction (0.4; *p* < 0.0001) and Rec increase (0.1; *p* = 0.0029) were statistically significant. Similarly, the mean number of sites with PD > 4 mm and absence of BoP significantly decreased between baseline and 6 weeks (−12.7; *p* < 0.0001). At this time point, the patient’s chewing discomfort was also significantly diminished (1.4; *p* = 0.0172). Conversely, no statistically significant changes were observed between 6 weeks and 3 months and between 3 months and 6 months for any of the clinical variables evaluated. In conclusion, within the limitation of this study, mechanical piezo-assisted non-surgical periodontal treatment in conjunction with an innovative tip resulted significantly efficacious to reduce pathological periodontal pockets, to gain clinical attachment and to reduce gingival inflammation.

## 1. Introduction

Periodontitis is a multifactorial inflammatory condition associated with the dysbiotic biofilm of dental plaque that represents one of the most prevalent non-communicable chronic diseases. Indeed, it globally affects about 40% of the adult population, with approximately 10% suffering from severe forms [1,2]. Periodontitis leads to progressive deterioration of tooth-supporting tissues and, if left untreated, can determine the tooth loss and, ultimately, masticatory dysfunction [3]. Such consequences could have an impact on the patient’s nutrition, quality of life and self-esteem, thus representing a critical socio-economic challenge for public health [4]. Nevertheless, periodontitis adversely affects systemic health, with particular regard to diabetes and cardiovascular diseases [5,6].

According to the Clinical Practice Guideline for the treatment of periodontitis developed by the European Federation of Periodontology, periodontal therapy should be performed in a step-wise approach [7]. The first step of therapy consists in oral hygiene instruction, control of local and systemic periodontal risk factors, and removal of supragingival biofilm and calculus. Subsequently, the second step involves the subgingival instrumentation, accomplished with or without local or systemic adjunctive therapies. The goal of periodontal therapy is to reach probing depths (PD) ≤ 4 mm without bleeding on probing (BoP), in order to prevent disease progression [8]. If this endpoint is not met at the time of reevaluation following the first two steps of therapy, a third one, which may consist in repeated subgingival instrumentation or periodontal surgery, could be provided [7].

With regard to step two of periodontal therapy specifically, from a clinical point of view, current evidence demonstrates that treatment outcomes do not depend on whether it is conducted in multiple sessions (e.g., quad-rant-wise) or in one or two stages of therapy over a period of 24 h (e.g., full-mouth protocol), and the data to support the cost-effectiveness of one modality over the other are weak [9]. Furthermore, it seems that the type of instruments (hand or powder, alone or in combination) does not have a strong impact on clinical endpoints either. Nevertheless, all types of instruments are technique sensitive and require specific training. At the same time, no data are yet available to show robust differences in postoperative sensitivity and treatment time based on the instrument used [9]. 

Histologically, since studies reporting that bacterial endotoxins or bacteria do not penetrate cementum and the removal of diseased cementum was not necessary for a successful second step of therapy, the focus has shifted to instruments capable of guaranteeing minimal removal by means of a less aggressive and less invasive action [10,11,12]. In fact, cement preservation is necessary for optimal periodontal health as well as for periodontal regeneration [13,14,15]. Furthermore, the removal of the cementum can eventually lead to the exposure of the dentinal tubules, lesions of the pulp and dentinal hypersensitivity [16]. In these terms, piezoelectric devices were shown to be superior to hand instruments in cement preservation and reduction in root surface roughness and damage [17].

Recently, a novel tip for piezoelectric devices has been developed, characterized by iron, rough, double nano-structural coated, corindone-treated structure. The roughness was not due to an additive material on its surface, but to a subtraction of metallic portions from its surface. An in vitro study using scanning electron microscopic analysis compared the above-mentioned insert to a conventional plain iron tip in root surface instrumentation, demonstrating that it had a higher performance in terms of root surface debridement [18]. However, the in vivo clinical results of using this innovative tip have never been investigated.

The aim of this study was to observe the clinical effect of a non-surgical treatment of periodontal patients using a piezoelectric power-driven device with a novel metallic insert.

## 2. Materials and Methods

The study was conducted at the Section of Periodontics of the Department of Oral and Maxillofacial Sciences of Sapienza, University of Rome, between March 2018 and June 2020. The present trial received the approval by the local ethical committee (Rif.. n.: 4909, prot. n.: 91/19; date: 1 February 2018). Informed consent was obtained from all subjects to be entered in the study. In obtaining the informed consent and in the conduct of the study the principles outlined in the Declaration of Helsinki on experimentation involving human subjects were adhered to.

### 2.1. Eligibility Criteria

The recruited patients had to satisfy the following inclusion criteria: (a) age ≥ 18 years; (b) presence of stage I to IV periodontitis, according to the 2018 classification case definition [19]; (c) presence of at least 3 teeth per quadrant with at least one site with PD > 4 mm in each quadrant.

Exclusion criteria were: (a) systemic diseases; (b) smoking; (c) pregnancy or lactating; (d) active periodontal treatment in the previous 6 months; (e) systemic antibiotic therapy in the previous 6 months.

### 2.2. Treatment Phase I: Oral Hygiene Instruction and Supragingival Instrumentation

After having been entered into the study, at the same appointment, all patients received:

- Appropriate oral hygiene instructions and motivation;
- Control of local and systemic periodontal risk factors;
- Full-mouth supragingival professional prophylaxis using ultrasonic/hand-instruments.

### 2.3. Treatment Phase II: Subgingival Instrumentation

Subgingival instrumentation was performed by a single operator (MC) with more than 20 years of experience in periodontal treatment.

**Figure 1:**
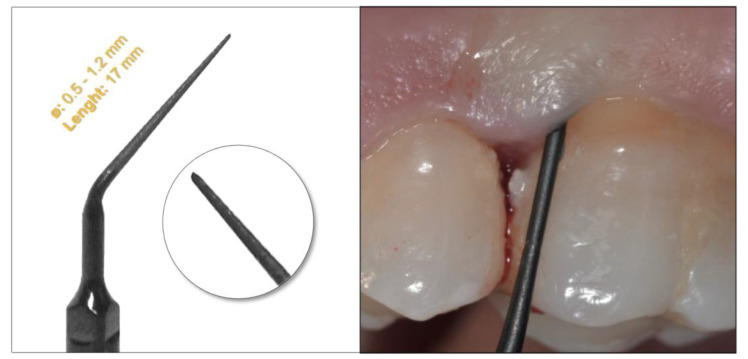
Graphic representation of the novel periodontal tip (**left**) and clinical photograph showing its use in subgingival instrumentation (**right**).

Scaling and root planing were performed using a full-mouth approach in all patients under local anesthesia using a piezoelectric device (Surgisonic Moto, Esacrom, Imola, Italy) associated with a novel periodontal tip characterized by a pointed shape and a round section (ES030ACT, Esacrom, Imola, Italy). The insert has a taper ranging from 0.5 to 1.2 mm, with a length of the working part of 17 mm. It is treated with T-COR technology and covered in T-BLACK. The T-COR is a treatment that involves the subtraction of material instead of the addition to make the surface of the insert rough. The subtraction of the metal has advantages during the treatment phase, as it allows for the procedure without the release of particles. The T-BLACK surface treatment allows for a reduction in tissue overheating and a considerable resistance to wear and corrosion. Another peculiarity is represented by the anti-reflection, which permits a better view of the operating field. Moreover, the dark color of the insert guarantees greater visibility when it is inside the pocket (Figure 1).

Periodontal therapy was performed in sites with a periodontal probing depth more than 3 mm until the operator felt a planed and well-debrided dental surface. 

Chair-time, recorded in minutes, was calculated since operator started with the procedure and after injection of local anesthesia.

Supragingival and subgingival instrumentation performed after phase 2 post lockdown due to SARS-CoV-2 pandemic followed the operative indications for health care professionals [20,21].

### 2.4. Post-Treatment Instructions

Patients were instructed to discontinue toothbrushing for 2 days, avoiding trauma at the treated sites. A 60 s rinse with 0.12% chlorhexidine digluconate was prescribed 2 times/day for this period. From the third to the seventh day, tooth cleaning by toothbrush and interproximal instruments restarted and the mouth rinse was discontinued. 

Patients were recalled for controls and supportive periodontal therapy (and prophylaxis as needed) at weeks 6, 12 and 24.

### 2.5. Clinical Measurements

One week after the instruction and oral hygiene procedures, the following baseline clinical data were collected by one blinded examiner (LM), different from the operator, using a calibrated periodontal probe (PCP UNC 15 Hu-Friedy):

- Probing depth (PD) at 6 sites for each tooth;
- Bleeding on probing (BoP) at 6 sites for each tooth, according to Ainamo and Bay [22];
- Plaque index (PlI) at 6 sites for each tooth, according to O’Leary et al. [23];
- Recession depth (Rec) at 6 sites for each tooth;
- Clinical attachment level (CAL) of the 6 sites per each tooth was calculated as PD + Rec (Rec was equal to 0 whenever the cementum-enamel junction was covered);
- Tooth mobility, according to the Miller’s classification [24];
- Furcation involvement, according to the Hamp et al. [25], evaluated with Naber’s probe;
- Full-mouth plaque score (FMPS) and full-mouth bleeding score (FMBS) [23].

The same clinical measurements were collected at the follow-up visits at 6 weeks, 3 months and 6 months.

The examiner underwent a training and calibration session on 5 patients not participating in the trial. He was asked to measure PlI, BoP, PD, Rec and CAL at six sites per tooth at two separate time intervals at least 120 min apart. The calibration was not considered acceptable if the intraclass correlation coefficient (ICC) was <0.81. 

### 2.6. Patient Reported Outcome Measures

At baseline, 6 weeks, 3 months and 6 months, questionnaires were administered to all patients, in order to record the chewing discomfort by means of a visual analogue scale (VAS) using a “from 0 to 10” scale, where “0” indicated “no discomfort”, and “10” “high discomfort”. Presence or absence of dental hypersensitivity was also recorded.

In addition, at each follow-up visit, the examiner recorded whether there were any adverse events related to the study procedures or whether any complications were reported by the patients.

### 2.7. Statistical Methods

Descriptive statistics were performed using mean and standard deviation for quantitative data and frequency and percentage for qualitative data.

Mixed-effect models were applied for CAL, PD, Rec, FMPS, FMBS, number of sites with PD > 4 mm and BoP+, number of teeth with hypermobility considering the patient as random effect and data recording time (baseline, 6 weeks, 3 months and 6 months) as fixed effect. Differences between the follow-ups were evaluated with the post-hoc Tukey HSD test.

The assumption of normality and homoscedasticity of the residuals of the statistical models were checked with graphical analyses.

All the statistical analyses were carried out using the same software (JMPs 13.0 Copyright SAS Institute Inc., SAS Campus Drive, Cary, NC, USA).

## 3. Results

Eighteen patients, 13 females and 5 males, with 55.1 ± 10.9 years of mean age (ranged between 25 and 74 years), providing 437 teeth (with a mean of 24.3 ± 2.4 teeth per patient), and 2622 sites in total were recruited in a consecutive way and considered eligible for the study.

**Figure 2:**
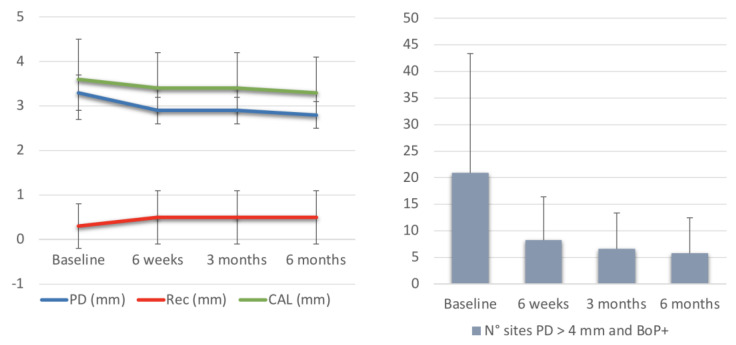
Graphic representation of mean values of PD, Rec, CAL and number of sites with PD < 4 mm and BoP+ at baseline, 6 weeks, 3 months and 6 months.

The baseline characteristics of the enrolled subjects revealed a mean CAL of 3.6 ± 0.9 mm, mean PD of 3.3 ± 0.6 mm, and a mean Rec of 0.3 ± 0.5 mm. The mean number of sites per patient with PD > 4 mm and associated BoP+ was 20.9 ± 22.5. In addition, 7 out of 18 subjects (39%) perceived discomfort/pain at baseline, but its intensity resulted quite low (with a mean pain VAS of 3.3 ± 3.9). Dental hypersensitivity was present in 5 out of 18 (28%) participants (Figure 2).

After 6 weeks, mean CAL and mean PD values decreased to 3.4 ± 0.8 and 2.9 ± 0.4 mm, respectively, while mean Rec increased to 0.5 ± 0.6 mm. Moreover, the mean number of sites per patient with PD > 4 mm and associated BoP+ decreased to 8.3 ± 8.1. Dental hypersensitivity increased in three subjects more (44%), but chewing discomfort (1.9 ± 2.9), FMPS (20.6 ± 10.7), FMBS (19.2 ± 11.3), and tooth mobility (1.7 ± 2.2) decreased significantly (Figure 2).

At 3-month assessment, mean CAL (3.4 ± 0.8), mean PD (2.9 ± 0.3), and mean Rec (0.5 ± 0.6) remained quite stable. FMPS, FMBS, chewing discomfort (1.5 ± 2.6) and dental hypersensitivity (17%) continued to decrease, although the changes were not statistically significant. The mean number of sites per patient with PD > 4 mm and associated BoP+ further decreased to 6.6 ± 6.8 (Figure 2).

At 6 months, mean CAL (3.3 ± 0.8), mean PD (2.8 ± 0.3), and mean Rec (0.5 ± 0.6) were almost unchanged, whereas FMPS (13.1 ± 4.6), FMBS (11.6 ± 6.7), chewing discomfort (1.6 ± 2.7) and dental hypersensitivity (11%) were slightly reduced. Mean number of sites per patient with PD > 4 mm and associated BoP+ was 5.8 ± 6.7 (Figure 2).

Overall, considering differences between different follow-ups, CALgain showed significant differences between baseline and 6 weeks (0.4; 95%CI 0.3; 0.6; *p* ≤ 0.0001) and baseline and 6 months (0.3; 95%CI 0.2; 0.4; *p* ≤ 0.0001). Similarly, PD difference was significant between baseline and 6 weeks (0.4; 95%CI 0.3; 0.6; *p* ≤ 0.0001) and baseline and 6 months (0.4; 95%CI 0.3; 0.6; *p* ≤ 0.0001). Recession reduction difference resulted significant between baseline and 6 weeks (−0.1; 95%CI −0.2; 0.0; *p* = 0.0029) and baseline and 6 months (−0.2; 95%CI −0.2; −0.1; *p* ≤ 0.0001). The mean difference of number of sites per patient with PD > 4 mm and associated BoP+ was significant between baseline and 6 weeks (12.7; 95%CI 4.5; 20.8; *p* ≤ 0.0001) and baseline and 6 months (15.1; 95%CI 7.0; 23.2; *p* ≤ 0.0001) (Table 1).

In addition, a significant difference was noticed for FMPS between baseline and 6 weeks (23.2; 95%CI 14.7; 31.7; *p* ≤ 0.0001) and baseline and 6 months (29.3; 95%CI 20.8; 37.8; *p* ≤ 0.0001). In terms of FMBS, significant differences were observed between baseline and 6 weeks (34.6; 95%CI 24.3; 44.9; *p* ≤ 0.0001) and baseline and 6 months (43.6; 95%CI 33.3; 53.9; *p* ≤ 0.0001) (Table 1).

Moreover, the chewing discomfort revealed significant differences between baseline and 6 weeks (1.4; 95%CI 0.2; 2.6; *p* = 0.0172) and baseline and 6 months (1.7; 95%CI 0.5; 2.9; *p* = 0.0020) (Table 1).

At the other follow-up steps, all the variables showed a continuous but not significant improvement, with the exception of tooth mobility that resulted unchanged.

The recorded mean time of execution of the treatment was 51.7 ± 25.2 min. 

No complications or adverse events related to the study device were observed or referred to by patients.

## 4. Discussion

The effectiveness of the second step of periodontal therapy as part of periodontal treatment is strongly supported by scientific evidence. However, most studies on this topic have been conducted in research settings with no potential confounders. In this context, manual instruments have produced smoother surfaces and greater removal of plaque and calculus [26], while ultrasonic devices require less time and are less dependent on the operator, in addition to removing less hard tissue structures from the tooth and cause less trauma to the periodontal soft tissue [27,28]. In clinical practice, hand instrumentation and ultrasound devices are often used in combination. Furthermore, it should be noted that with regards to the clinical endpoint, a recent systematic review did not reveal any significant differences between these two treatment modalities [9].

There are many different protocols that include innovative materials and tools and/or adjunctive therapies to be used in step two, but they have not yet been fully validated. A variety of different instrumentations in terms of manufacturer, design, and technology have been proposed, and researchers have mainly focused their attention on identifying less time-consuming therapeutical approaches showing an even greater efficacy in terms of improved clinical outcomes [29]. In 2018, Rotundo et al. reported data from an in vitro study comparing a novel iron, rough, double nano-structural coated corindone-treated tip (T-Black) with a conventional iron smooth tip [18]. Twenty freshly extracted teeth were collected (10 per group) and a total surface of 21.4 × 106 mm^2^ was analyzed by scanning electronic microscope. The percentage of residual calculus after ultrasonic scaling in Test Group was 1.9 ± 1.8%, while in Control Group it was 5.7 ± 4.3%, with a significant difference between the experimental groups (*p* = 0.019). The present study reported for the first time the clinical results from a series of periodontal patients treated by means of a piezoelectric-assisted machine and the above-mentioned tip. 

The most frequently selected treatment outcome to evaluate the efficacy of the second step of periodontal therapy is the reduction in PD. Indeed, PD is considered a surrogate outcome variable of periodontitis progression and has been associated with tooth loss by several studies [30]. In the present investigation, the mean initial probing depth was 3.3 ± 0.6 mm, and 6 months after treatment the mean reduction in pocket depth achieved was 0.4 ± 0.4 mm (*p* ≤ 0.0001). These changes were accompanied by a slight increase in gingival recession (0.2 mm ± 0.2; *p* ≤ 0.0001), allowing for a final CAL gain of 0.3 ± 0.2 mm (*p* ≤ 0.0001). Parameters related to periodontal inflammation and plaque accumulation also decreased during the study. It is noteworthy to observe how these changes could already be observed after 6 weeks, while no significant improvements occurred in the subsequent time intervals. Taken together, these variations are in line with other clinical studies and systematic reviews dealing with subgingival mechanical instrumentation in patients with different baseline conditions [31,32,33,34,35].

Nevertheless, the goal of treatment is to achieve sufficient biofilm and calculus removal and consequently resolve the inflammatory condition. Therefore, pocket closure, understood as shallow probing pocket depth and absence of bleeding, has been nowadays considered the most important component to determine the efficacy of the therapy. Regarding this outcome measure, the present study recorded significant improvements between baseline and 6 weeks that remained fairly stable through the 6-month follow-up. The 72.2% increase in pocket closure observed in this investigation was similar to that described by Suvan et al. [9], who recently reported a 74% (95% CI 64–85) reduction in probing pocket depth sites > 4 mm with the presence of bleeding after conventional nonsurgical therapy. Conversely, the data presented here performed better than those reported in a recent systematic review by Citterio et al. [36], where the mean number of residual pockets after conventional nonsurgical therapy performed with sonic/ultrasonic and manual instruments was higher (14.13 vs. 5.8). This finding could be partly explained by the moderate severity of periodontitis in the patients treated in the present investigation. Indeed, it has been shown that in less advanced cases, resolution of the disease, as measured by pocket closure, is more likely [9].

Concerning the PROMS, the investigated treatment provided a significant reduction in masticatory discomfort assessed through a VAS scale (1.7; 95%CI 0.5–2.9; *p* = 0.0020). This improvement was accompanied by an increase in three (16%) subjects experiencing dentin hypersensitivity. However, this event was only temporary and was barely superior to a similar study using full-mouth ultrasonic debridement in which this symptom affected only 5% of treated patients [37].

Interestingly, time spent by the operator for subgingival instrumentation in this study averaged just under 60 min. This result is not surprising considering that satisfactory clinical results in the initial treatment of patients with periodontitis can be achieved by a 1 h session of whole-mouth subgingival debridement using a piezoceramic ultrasonic instrument [37].

Undoubtedly, this study has limitations. Among these, the design of the trial must be considered. In fact, there was not a control group and the results could not be compared with those achievable with a gold-standard therapy. Furthermore, the study follow-up ends at 6 months, which could be considered short. However, it should be noted that the second step of therapy is part of an overall therapeutic strategy where the 6-month follow-up interval could be considered appropriate for clinical re-evaluation and most studies in this field rarely extend beyond this time point [9]. Furthermore, PROMS could have been further studied using validated questionnaires for assessing oral related quality of life. Finally, biochemical and microbiological analyses are suggested for future randomized controlled studies.

## 5. Conclusions

Within the limits of the study, the use of the iron, rough, double nano-structural coated, corindone-treated tip with a calibrated superficial roughness working on piezo-electric machine in the second step of periodontal therapy led to a reduction in pathological periodontal pockets, a gain of clinical attachment and a decrease in gingival inflammation.

Further studies are needed to substantiate the efficacy of the tested device compared to conventional hand instruments and ultrasound devices with or without adjunctive therapy.

## Figures and Tables

**Table 1:** Clinical variable differences between different follow-ups.

Variables	Estimate	95%CI	*p*-Value
**CAL** (mm)			
Baseline—6 months	0.3	0.2; 0.4	<0.0001
Baseline—6 weeks	0.4	0.3; 0.6	<0.0001
6 weeks—3 months	0.1	−0.1; 0.2	0.6751
3 months—6 months	0.0	−0.1; 0.2	0.9314
**PD** (mm)			
Baseline—6 months	0.4	0.3; 0.6	<0.0001
Baseline—6 weeks	0.4	0.3; 0.6	<0.0001
6 weeks—3 months	0.1	−0.1; 0.2	0.6751
3 months—6 months	0.0	−0.1; 0.2	0.9314
**Rec** (mm)			
Baseline—6 months	−0.2	−0.2; −0.1	<0.0001
Baseline—6 weeks	−0.1	−0.2; −0.0	0.0029
6 weeks—3 months	−0.0	−0.1; 0.1	0.7874
3 months—6 months	−0.0	−0.1; 0.1	0.9734
**FMPS** (%)			
Baseline—6 months	29.3	20.8; 37.8	<0.0001
Baseline—6 weeks	23.2	14.7; 31.7	<0.0001
6 weeks—3 months	4.1	−4.4; 12.6	0.5730
3 months—6 months	2.0	−6.5; 10.5	0.9220
**FMBS** (%)			
Baseline—6 months	43.6	33.3; 53.9	<0.0001
Baseline—6 weeks	34.6	24.3; 44.9	<0.0001
6 weeks—3 months	6.4	−3.9; 16.7	0.3665
3 months 6 months	2.6	−7.7; 12.9	0.9049
**N° sites with PD > 4, BoP+**			
Baseline—6 months	15.1	7.0; 23.2	<0.0001
Baseline—6 weeks	12.7	4.5; 20.8	0.0001
6 weeks—3 months	1.7	−6.4; 9.9	0.9427
3 months—6 months	0.7	−7.4; 8.9	0.9953
**Tooth mobility** (n°)			
Baseline—6 months	1.0	−0.4; 2.4	0.2559
Baseline—6 weeks	1.0	−0.4; 2.4	0.2559
6 weeks—3 months	0.0	−1.4; 1.4	1.0
3 months—6 months	0.0	−1.4; 1.4	1.0
**Chewing Discomfort** (VAS)			
Baseline—6 months	1.7	0.5; 2.9	0.0020
Baseline—6 weeks	1.4	0.2; 2.6	0.0172
6 weeks—3 months	0.4	−0.8; 1.6	0.8246
3 months—6 months	−0.1	−1.3; 1.1	0.9993

## Data Availability

The data presented in this study are available on request from the corresponding author.
